# Effect of the administration of a fermented milk containing *Lactobacillus casei *DN-114001 on intestinal microbiota and gut associated immune cells of nursing mice and after weaning until immune maturity

**DOI:** 10.1186/1471-2172-9-27

**Published:** 2008-06-13

**Authors:** Alejandra de Moreno de LeBlanc, Cecilia A Dogi, Carolina Maldonado Galdeano, Esteban Carmuega, Ricardo Weill, Gabriela Perdigón

**Affiliations:** 1 Centro de Referencia para Lactobacilos (CERELA-CONICET), Chacabuco 145, San Miguel de Tucumán (T4000ILC) Tucumán, Argentina; 2 Cátedra de Inmunología, Instituto de Microbiología, Facultad de Bioquímica, Química y Farmacia, Universidad Nacional de Tucumán, Argentina; 3 Nutritia. Buenos Aires, Argentina; 4 Departamento de investigación y Desarrollo, DANONE Argentina S.A. Buenos Aires, Argentina

## Abstract

**Background:**

Microbial colonization of the intestine after birth is an important step for the development of the gut immune system. The acquisition of passive immunity through breast-feeding may influence the pattern of bacterial colonization in the newborn. The aim of this work was to evaluate the effect of the administration of a probiotic fermented milk (PFM) containing yogurt starter cultures and the probiotic bacteria strain *Lactobacillus casei *DN-114001 to mothers during nursing or their offspring, on the intestinal bacterial population and on parameters of the gut immune system.

**Results:**

Fifteen mice of each group were sacrificed at ages 12, 21, 28 and 45 days. Large intestines were taken for determination of intestinal microbiota, and small intestines for the study of secretory-IgA (S-IgA) in fluid and the study of IgA+ cells, macrophages, dendritic cells and goblet cells on tissue samples. The consumption of the PFM either by the mother during nursing or by the offspring after weaning modified the development of bifidobacteria population in the large intestine of the mice. These modifications were accompanied with a decrease of enterobacteria population. The administration of this PFM to the mothers improved their own immune system and this also affected their offspring. Offspring from mice that received PFM increased S-IgA in intestinal fluids, which mainly originated from their mother's immune system. A decrease in the number of macrophages, dendritic cells and IgA+ cells during the suckling period in offspring fed with PFM was observed; this could be related with the improvement of the immunity of the mothers, which passively protect their babies. At day 45, the mice reach maturity of their own immune system and the effects of the PFM was the stimulation of their mucosal immunity.

**Conclusion:**

The present work shows the beneficial effect of the administration of a PFM not only to the mothers during the suckling period but also to their offspring after weaning and until adulthood. This effect positively improved the intestinal microbiota that are related with a modulation of the gut immune response, which was demonstrated with the stimulation of the IgA + cells, macrophages and dendritic cells.

## Background

The gastrointestinal tract (GIT) of adult mammals is colonized by a complex and dynamic community of microorganisms in a process of natural selection and ecological succession. The composition of this microbiota depends on various factors, some of which are of host origin, such as the genome and physiology of the animal, whereas others are of microbial origin, such as interactions between bacterial species [1]. After birth, the germ-free human GIT is rapidly colonized by facultative anaerobic bacteria (e.g., Enterobacter) that encourage the growth of anaerobic bacteria such as lactobacilli; bifidobacteria; Bacteroides and clostridia [2,3]. At weaning, with the introduction of solid food and deprivation of their mother's milk, the young must also cope with a rapidly changing microbiota. This is a stress time where, according to Ewing and Cole [4], numbers of lactobacilli and other beneficial bacteria could decrease as do their beneficial effects, allowing potential pathogens such as coliforms to expand.

The resident intestinal microbiota confers many benefits to the host [5]. Some of these benefits include the metabolism of nutrients and organic substrates, and the contribution to the phenomenon of colonization resistance [6].

In experimental studies, the role of the microbiota is determined by comparing germ free and conventional animals; newborn germ-free animals exhibit an underdeveloped intestinal immune system. Experiments using gnotobiotic animals have shown that association of germ-free rodents with a single bacterial specie has a profound impact on the anatomical, physiological, and immunological development of the host. This includes microbicidal protein production, development of intestinal epithelium; vasculature and gut associated lymphoid tissue (GALT) [7-9].

The beneficial effects of the microbiota on the immune system have been proposed as a theory supporting the use of non pathogenic bacteria, including probiotics in improving animal health and protection against infectious agents [10]. Probiotics are live microorganisms which, when administered in adequate amounts confer a health benefit on the host [11]. These microorganisms can influence the composition and activity of the gut microbiota, modulate the inflammatory response, improve the non-specific intestinal barrier, and reinforce or modulate the mucosal and the systemic immune response [12]. There are many reports about the beneficial effect of the consumption of fermented milk containing the probiotic strain *Lactobacillus casei *DN-114001 [13-15]. It was observed that long term fermented milk administration had immunodulatory effect and maintained the intestinal homeostasis without adverse secondary effects in mice [13].

During the early phases of development, neonates (human or mice) not only rely on their own innate immune system to help combat infections, they also acquire adaptive and innate immunity through maternal sources (via transplacental routes and breast milk), a process collectively referred to as passive immunity. Passive immunity provides a number of defense factors such as immunoglobulins, lactoferrin, lysozyme, oligosaccharides, cytokines, and chemokines [16,17]. Passive immunity may also influence the development of the systemic and mucosal adaptive immune system of newborn mice [18].

The aims of this study was to investigate, using a mouse model, how the administration of a fermented milk containing the probiotic bacteria *L. casei *DN-114001, whose immunomodulatory capacity in adult conventional mice was demonstrated [13], may affect the composition of the intestinal bacterial population and influence the intestinal non specific barrier, or the immune cells associated to the gut, involved in the innate immunity, in newborn mice before and after weaning. The present study was designed to evaluate both the consumption of probiotic fermented milk by the mother and the effect on their offspring during the suckling period and the supplementation to the newborn's diet with this fermented milk after weaning.

## Methods

### Animals and protocol design

**Figure 1:**
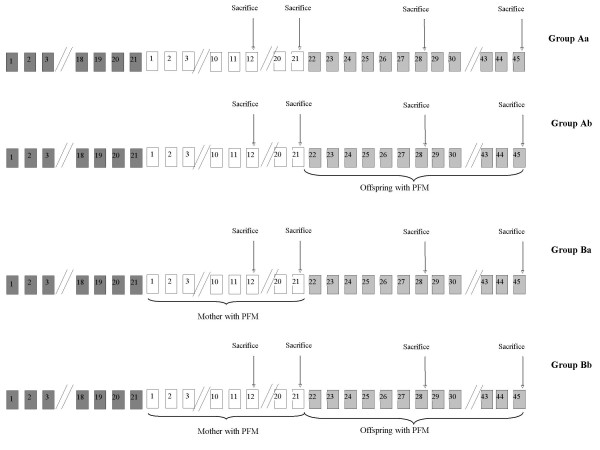
**Design of the different experimental groups under study**. Dark gray square are used for the pregnancy period (21 days approximately); white square are used for the suckling period (21 days) and light gray square are used for the period after weaning (21 days of age) and until adulthood (45 days of age). Arrows indicate the day of the sacrifice (12, 21, 28 and 45 after birth). Brackets are used to indicate the periods where the mothers or their offspring receive the PFM.

BALB/c mice used in this study were obtained from the closed random bred colony maintained at the CERELA (Centro de Referencia para Lactobacilos, San Miguel de Tucumán, Argentina). All the animals were fed a conventional balanced diet (23% proteins, 6% raw fiber, 10% total minerals, 1.3% Ca, 0.8% P, 12% moisture and vitamins) *ad libitum*. Pregnant mice (7 weeks old weighing 25–30 g) were identified and monitored daily until delivery. The day of birth was identified as day 0 of life. Babies were weaned at 21 days of age and the study was carried out until day 45. The experimental protocol contained two experimental groups of mothers: one receiving the fermented milk containing the probiotic strain *L. casei *DN-114001 (PFM) during the suckling period (B) and the second did not (A). At weaning, in both groups of mothers, the babies were divided into two subgroups: (b) those receiving PFM and (a) those that did not. Figure 1 describes the experimental design.

Babies were sacrificed by cervical dislocation at 12, 21, 28 and 45 days of age and samples of small and large intestine were obtained for immunological and microbiological studies.

All animal protocols were pre-approved by the Animal Protection Committee of CERELA and all experiments comply with the current laws of Argentina.

### Fermented milk and feeding procedure

Commercial fermented milk containing the yogurt starter cultures (*L. delbrueckii *subsp. *bulgaricus *10^8 ^CFU/ml and *Streptococcus thermophilus *10^8 ^CFU/ml) and the probiotic bacterium *L. casei *DN-114 001 (10^8 ^CFU/ml) was used in this study.

Mothers from the test group B received the commercial product *ad libitum *during the nursing period. After weaning, according to the protocol detailed above, offspring, from both A and B groups, received the same PFM or water continuously until day 45 of age *ad libitum*. They were the groups Aa, Ab, Ba and Bb, the first group (Aa) being the control group with no administration of PFM by the mothers or their offspring.

### Microbiology

The large intestines were aseptically removed, weighed and placed into sterile tubes containing 5 ml of peptone water (0.1%). The samples were immediately homogenized under sterile conditions using a homogenizer (MSE, England). Serial dilutions of the homogenized samples were obtained and aliquots (0.1 ml) of the appropriate dilution were spread onto the surface of following agarized media: Reinforced Clostridial (RCA, Britania, Buenos Aires, Argentina) for total anaerobic bacteria; RCA containing 0.2% LiCl, colistin 4 mg/l, 1% aniline blue and after sterilization adjusted to pH 5 with acetic acid (RCA-pH5) for isolation of bifidobacteria; Mann-Rogosa-Sharp Agar (MRS Britania, Buenos Aires, Argentina) for total lactobacilli; M-17 with colistin 4 mg/l (Difco, Elancourt, France) for lactic flora and Mac Conkey (Britania, Buenos Aires, Argentina) for Enterobacteriaceae. This last culture media was aerobically incubated at 37°C for 24 h, all others plates were anaerobically incubated at 37°C for 72 – 96 h.

### Immunofluorescence assay for IgA+ cells in small intestine

The tissues (small intestine) from the offspring were prepared for histological studies, fixed in formaldehyde, dehydrated using a graded series of ethanol and xylene, and embedded in paraffin following standard methodology.

The number of IgA positive cells was determined on histological slices using a direct immunofluorescence assay. After deparaffinization using xylene and rehydration in a decreasing gradient of ethanol, paraffin sections (4 μm) were incubated with a 1:100 dilution of α-chain monospecific antibody conjugated with FITC (Sigma, St Louis, MO, USA) for 30 min and observed with a fluorescent light microscope. The number of fluorescent cells was counted in 30 fields at 1000× magnification and results were expressed as the number of positive fluorescent cells per ten fields of vision.

### Secretory IgA in intestinal fluid

Intestinal fluid was collected from the small intestines of offspring mice in 1 ml of 0.85% NaCl, centrifuged at 5000 *g *during 15 min at 4°C, using a refrigerated centrifuge (Presvac, Buenos Aires, Argentina). The supernatant was recovered and stored at -20°C until IgA determination.

ELISA was used to measure the concentration of total S-IgA according to the technique described by LeBlanc et al [19]. Affinity-purified monoclonal goat anti-IgA (α-chain specific Sigma, St Louis, MO, USA) was added at 1.25 μg/well in 0.05 M carbonate-bicarbonate buffer (pH 9.6) to Costar 96-well, U-bottomed, high-binding polystyrene microplates (Nunc Inc.) and incubated at 37°C for 1 h. The plates were then washed three times with PBS containing 0.05% Tween 20 (PBS-T) and blocked for 1 h at 25°C with 0.5% nonfat dry milk in PBS. Plates were washed five times with PBS-T and incubated for 2 h at 37°C with either 50 μl of standard kappa IgA (Sigma, St. Louis, USA) or 50 μl samples of intestinal fluid in triplicate. Plates were washed seven times with PBS-T and incubated in the presence of horseradish peroxidase-conjugated anti-IgA-specific antibodies (Sigma, St. Louis, MO, USA) at 1.25 μg/well for 1 h at 37°C. Plates were again washed seven times, and 100 μl of trimethylbenzidine reagent containing peroxide (BD Biosciences, San Diego, CA, USA) was added to each well. Reactions were terminated with 100 μl of H_2_SO_4 _(2 N) with gentle shaking. The optical density was read at 450 nm using a VERSA Max Microplate Reader (Molecular Devices, USA).

### Determination of macrophage and dendritic cells in lamina propria of the small intestine

Macrophages were determined using the BM8 monoclonal antibody (Affinity Purified anti-mouse F4/80 Antigen – Pan Macrophage Marker, eBioscience, San Diego, CA, USA), which reacts with mouse F4/80 antigen. Dendritic cells were determined using the 33D1 monoclonal antibody (Affinity Purified anti-mouse Dendritic Cell Marker (33D1) eBioscience, San Diego, CA, USA) which recognizes a mouse dendritic cell-specific surface marker.

The tissues were treated as was previously described. After deparaffinization, slides from the different groups analyzed were incubated with a 1:50 dilution of primary antibody during 60 min at room temperature. Then the slices were washed twice in PBS and incubated for 45 min with a 1:100 dilution of the goat anti-rat antibody conjugated with FITC (Jackson Immuno Research Labs Inc, West Grove, USA) at room temperature and washed twice in PBS. The number of fluorescent cells was counted in 30 fields at 1000× magnification and results were expressed as the number of positive fluorescent cells per ten fields of vision.

### Determination of goblet cells in small intestine

Slides from the small intestine of the different groups under study, were deparaffinized and rehydrated in a decreasing gradient of ethanol and incubated for 150 min in 1% Alcian Blue 8Gx solution (Merck, Darmstadt, F.R. Germany) in 3% acetic acid. Histological slides were then incubated for 6 min in eosin solution and then 40 min in 0.5% safranin solution in 0.1 N HCl. They were then dehydrated and finally mounted using synthetic Canada Balsam (Ciccarelli Lab., San Lorenzo, Argentina). Goblet cells were stained blue with this methodology. The results are expressed as the number of goblet cells per ten intestinal villous.

### Statistical analysis

Statistical analysis were performed using MINITAB 14 software (Minitab, Inc., State College, PA, USA) by ANOVA GLM followed by a Tukey's posthoc test, and *P *< 0.05 was considered significant. Unless otherwise indicated, all values (n = 15) were the means of 3 independent trials (no significant differences were observed between individual replicates) ± standard deviation (SD).

## Results

### Effect of the PFM on the intestinal bacterial population during development

**Figure 2:**
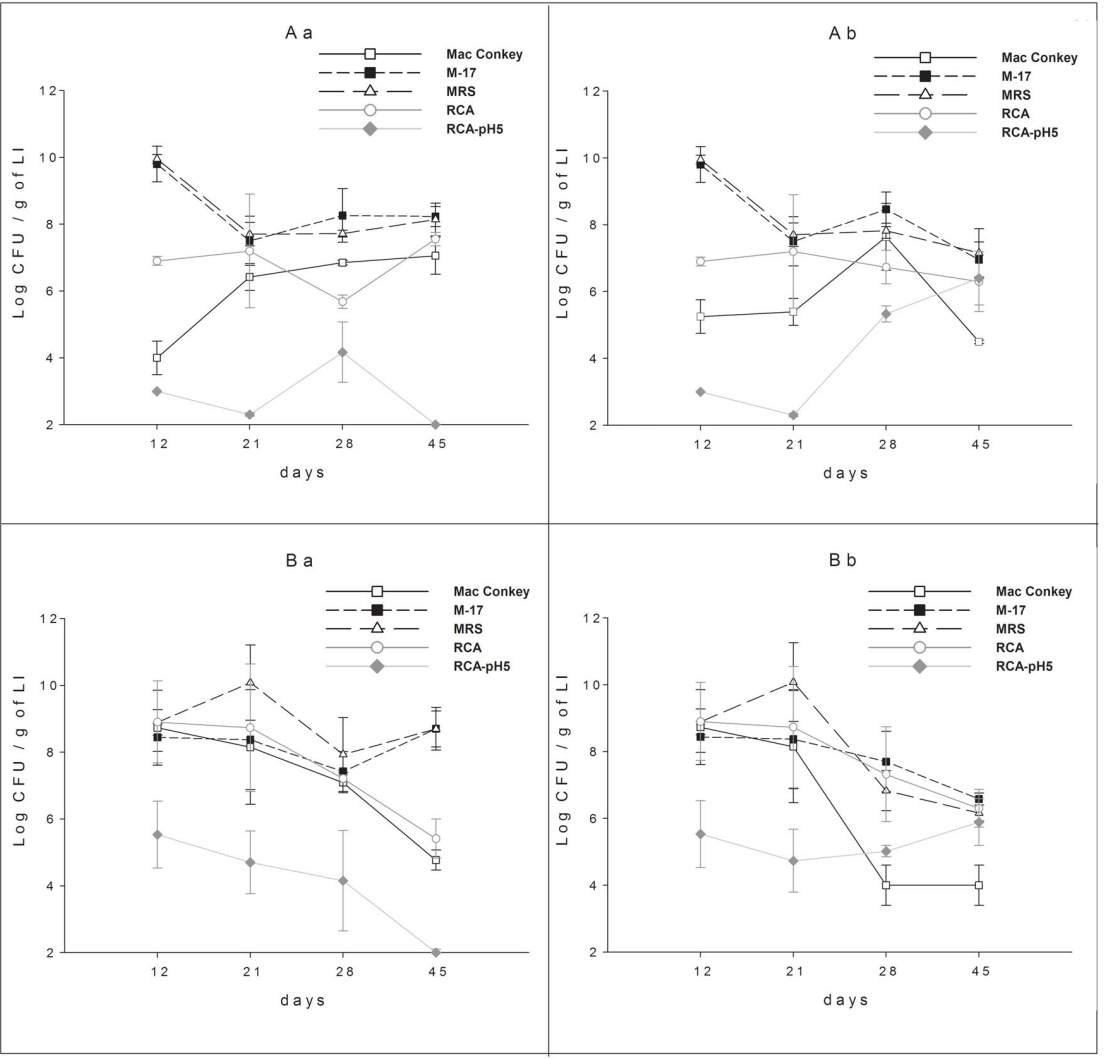
**Intestinal microbiota of the large intestine of newborn mice**. The large intestine were aseptically removed, weighed and placed into sterile tubes containing peptone water. The samples were immediately homogenized under sterile conditions and serial dilutions of the homogenized samples were obtained and aliquots of the appropriate dilution were spread onto the surface of following agarized media: Mac Conkey were used for enterobacteria; M-17 for lactic flora; RCA-pH5 for bifidobacteria; MRS for total lactobacilli; RCA total anaerobic bacteria. Colony counts are expressed as log_10 _numbers of bacteria per gram of large intestine. Each point represents the mean of n = 15 ± SD. In order to simplify the analysis of the figure, the statistical analysis is showed only for the two media where significant differences were observed (Mac Conkey and RCA-pH5) comparing all the groups. ^a,b^Means for each culture medium without a common letter differ significantly (*P *< 0.05).

The results obtained after the administration of PFM to the mothers during the suckling period or their offspring after weaning, showed that the most remarkable differences were found between enterobacteria and bifidoacteria. Mice born from mothers that received PFM during nursing (group B) showed a significant increase in the bifidobacteria counts mean log CFU (5.57 ± 0.5 for 12 days and 4.9 ± 0.4 for 21 days; Fig 2) compared with those from mothers that never received PFM (group A, 3.0 ± 0.1 and 2.3 ± 0.1, respectively for 12 and 21 days). At the same time, enterobacteria population was increased (3 log and 2 log for 12 and 21 days, respectively) in the mice coming from group B compared with those from group A. After weaning, mice of group Ba showed a progressive diminution in the bifidobacteria population, with values similar to those the control group (Aa). The same was observed for the enterobacteria comparing these groups of mice (Fig. 2). In contrast, after 28 days, newborns from group Ab had higher numbers of bifidobacteria. Reaching values similar to those of the group that received PFM during all the experiment (Bb) and, for this group (Ab), enterobacteria decreased when bifidobacteria increased (Fig. 2). At the same period of time, mice from group Bb had bifidobacteria concentrations similar to the nursing and the enterobacteria counts diminished significantly (Fig. 2)

No significant changes were observed for the other bacteria studied comparing different groups; only an increase of anaerobic bacteria mean log CFU (8.9 ± 1.2) was observed in the mice from group B at day 12, compared to mice from group A (6.9 ± 0.1), Fig. 2.

### Influence of the PFM administration on IgA+ cells of the small intestine and total S-IgA levels

**Figure 3:**
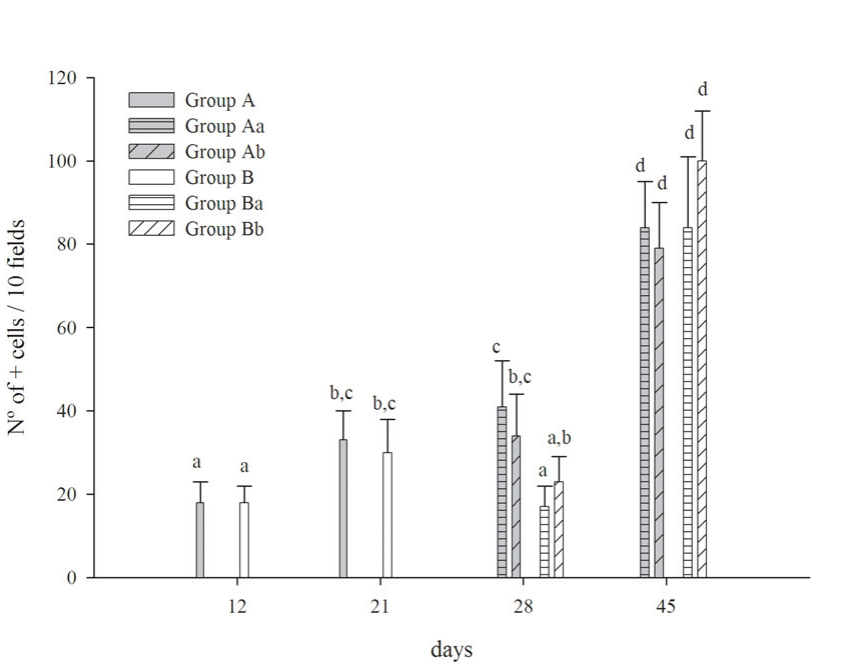
**IgA+ cells in the small intestine of newborn mice**. IgA+ cells were determined by direct immunofluorescence on slides from small intestine of mice of different ages of life (12, 21, 28 and 45 days). Results are expressed as number of positive cells per 10 fields of vision at 1000× of magnification. Values are means for n = 15 ± SD. Mice from each exprimental group (mice from mother did not received PFM and they did not receive PFM (Aa) or they received PFM after weaning (Ab); mice from mothers given PFM during suckling period and they did not receive PFM (Ba) or they received PFM (Bb) after weaning. Means for each value without a common letter differ significantly (*P *< 0.05).

At 12 days of age, the IgA + cells in the small intestine of the newborn mice did not show significant differences between newborn mice whose mothers did or did not receive PFM. At weaning and in the samples on days 28 and 45, newborns from control group (Aa) showed a progressive increase of IgA+ cells. In contrast, mice from group B showed a lower count of IgA+ cells at day 28. At the end of the experiment (45 days) all the groups showed similar values for these cells (Fig. 3), independent of the consumption of PFM by the newborns.

**Figure 4:**
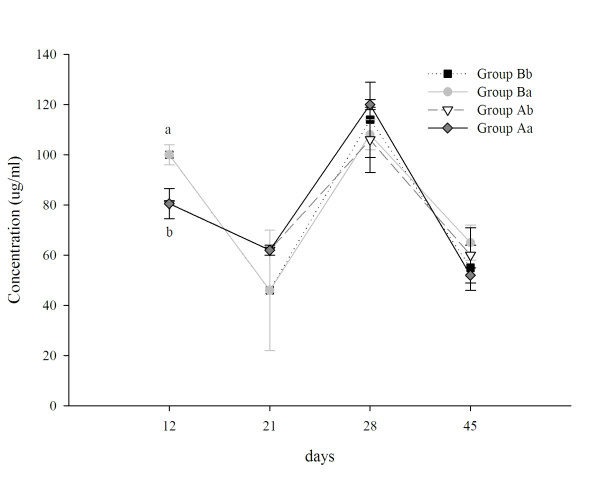
**S-IgA in the small intestinal fluid of newborn mice**. ELISA was used to measure the concentration of total S-IgA in the small intestine fluid obtained from mice of different experimental groups and at different time point. Results are expressed as concentration (μg/ml). Each point represents the mean of n = 15 ± SD mice from each group (Aa: mother and offspring without PFM; Ab: mother without PFM and offspring with PFM after weaning; Ba: mother with PFM and offspring without PFM after weaning; Bb: mother with PFM and offspring with PFM after weaning). In order to simplify the figure, the statistical analysis is showed only for the first time point where significant differences (*P *< 0.05) were observed between mice from groups Ba and Bb and mice from groups Aa and Ab. ^a,b ^Different letters are used to show the significantly differences.

At day 12 of age, mice from group B showed a significant increase of total S-IgA in the intestinal fluid compared to group A. On day 21 the values decreased in all the groups with no observable differences among the assayed groups (Fig. 4). For day 28, the values were the highest in all the groups; however, no significant differences among each group were observed. In the adult period (45 days) PFM did not influence these values (Fig. 4) compared to the control (Aa).

### Influence of PFM consumption on macrophages and dendritic cells of lamina propria of small intestine

Macrophages were determined using the BM8 monoclonal antibody which reacts with mouse F4/80 antigen, a transmembrane protein of approximately 125 kDa expressed by the majority of mature macrophages, which is currently the best marker for this cell population. The results obtained for F4/80+ cells showed that PFM consumption by mothers decreased macrophage numbers in the newborn mice at day 12 in respect to the group without PFM. At 45 days of age (adult mice) all the groups fed with PFM after weaning (Bb and Ab) showed a significant increase in the number of cells expressing F4/80 related to the group Ba and the control group Aa (Table 1).

**Table 1:** Counts of macrophages and dendritic cells of the small intestine.

**Experimental groups**			Macrophages	Dendritic cells
**Nursing**				

12 days	Group A		45 ± 12^b^	37 ± 12^b^
	Group B		24 ± 7^a,c^	16 ± 4^a^
21 days	Group A		22 ± 6^a,c,d^	12 ± 7^a,c^
	Group B		25 ± 8^a,c^	12 ± 3^a,c^

**Weaning**				

28 days	Group A	Aa	22 ± 7^a,c,d^	9 ± 2^c^
		Ab	14 ± 3^d^	11 ± 2^a,c^
	Group B	Ba	23 ± 7^a,c,d^	20 ± 7^a^
		Bb	18 ± 4^c,d^	16 ± 6^a,c^
45 days	Group A	Aa	32 ± 6^a^	14 ± 4^a^
		Ab	72 ± 10^e^	24 ± 3^b^
	Group B	Ba	45 ± 13^b^	18 ± 2^a^
		Bb	56 ± 10^b,e^	27 ± 6^b^

Macrophages and dendritic cells were determined by indirect immunofluorescence on the small intestine tissue slides of mice from different experimental group (Mother during suckling period without PFM (A) or with PFM (B) and offspring after weaning without PFM (a) or with PFM (b). Macrophages were determined using the BM8 monoclonal antibody which reacts with mouse F4/80 antigen and dendritic cells were determined using the 33D1 monoclonal antibody which recognizes a mouse dendritic cell-specific surface marker.

Results are expressed as number of positive cells recognized for the respective primary antibody, counted in 10 fields of vision at 1000× of magnification. Values are means for n = 15 ± SD mice from each group and at each time point. ^a,b,c,d,e ^Means for each cell population without a common letter differ significantly (*P *< 0.05).

Dendritic cells were determined using the 33D1 monoclonal antibody which recognizes a mouse DC-specific surface marker. The nature and biological activity of the 33D1 antigen has not yet been elucidated. 33D1 has been found on a variety of dendritic cell subpopulations from mouse thymus, spleen, lymph node, and Peyer's patch.

The pattern obtained for dendritic cells was similar to the macrophages, we observed a decrease in the number of cells recognized by 33D1 antibody in mice from group B at day 12 in comparison with group A. At day 45, it was observed that dendritic cell numbers were increased by the consumption of the PFM in mice from groups Bb and Ab (Table 1).

### Effect on the nonspecific barrier: goblet cells determination

**Figure 5:**
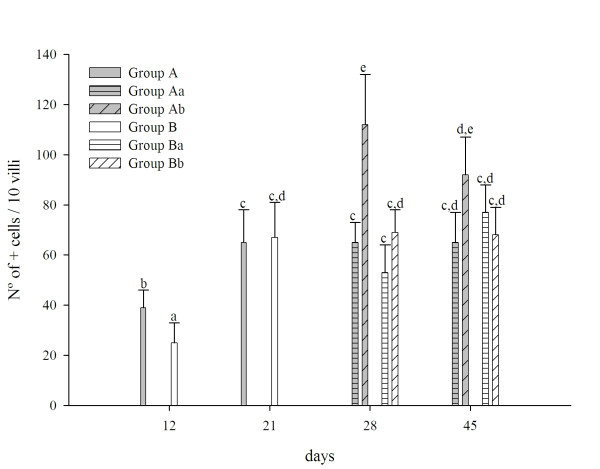
**Goblet cells in the small intestine of newborn mice**. Goblet cells were stained with alcian blue on **s**lides from the small intestine of mice from different groups under study. Values are means for n = 15 ± SD mice. ^a,b,c,d,e ^Means for each value without a common letter differ significantly (*P *< 0.05).

The number of goblet cells decreased in the first sample (12 days) in newborn mice from group B (Fig. 5). At 21 days of age, both groups of mice reached similar values to those observed in the control adult mice (65 ± 12, group Aa). After weaning, on the 28 and the adult age (45 days), the effect of the consumption of PFM by the offspring was only observed in the offspring of from mothers that never received PFM (Ab).

## Discussion

The concentration of the bacterial species in the intestinal tract varied with the age and with the diet of the mice. There are many reports that showed the relationship between the administration of fermented milk containing probiotic bacteria and the increase in *Bifidobacterium *numbers [20,21]. This can be related with the recent observation that some probiotic strains posses metabolic pathways needed for the synthesis and release of molecules that selectively stimulate the growth of endogenous bifidobacteria. It has been suggested that the increase in the concentration of these bacteria could confer a beneficial effect on the stability of the intestinal microbiota [22]. Other reports have demonstrated the beneficial effects of oral administration of probiotic bacteria on the intestinal microbiota, especially after antibiotic therapy [23] or in stress conditions such as malnutrition [24]. Using experimental murine models of malnutrition, it was suggested that the ingestion of *L. casei *CRL 431 or conventional yoghurt was able to restore the gut microbiota [25]. In these studies either the bacteria or yoghurt were able to recover the equilibrium between aerobic and anaerobic strict bacteria. This previous knowledge and the demonstration of the immunomodulatory capacity of yoghurt [26,27] and a fermented milk containing the probiotic bacteria DN-114001 [13], led us to analyze the effect of a PFM administered to the mothers during suckling period or to their offspring (after weaning) on their microbiota and in some parameters of the gut immune function such as the nonspecific barrier, IgA+ B lymphocytes, macrophages and dendritic cells.

The PFM was selected due to the presence of a probiotic bacterium and the biological active components produced during fermentation such as peptides and carbohydrates which are able to influence both the indigenous microbiota and the host immune function. In previous studies the importance of the nonbacterial fraction from milk fermented by *L. helveticus *R389 in the immune stimulation under normal or pathological conditions (intestinal infection, breast cancer) was evaluated [28,29].

In the present work, it was observed that the consumption of fermented milk containing *L. casei *DN-114 001, either by the mother mice during nursing period or by their offspring after weaning, influenced the development of the bifidobacteria population in the large intestine of the newborn. These increases were accompanied with a decrease of enterobacteria population (Fig. 2). These findings agree with the results obtained by other authors where they evaluated the effect of probiotic consumption on the intestinal microbiota composition: bifidobacteria were increased and the concentration of enterobacteria and *Clostridium *decreased [30,31]. The decrease of this latter microbial population is desirable since *E. coli *and *Clostridium *have been implicated in the production of amonium, aminas and some carcinogens [32]. In contrast, many beneficial effects were attributed to bifidobacteria [33]. *Bifidobacterium *has been related with the regulation of oral tolerance [34]. With respect to the effect of the microbiota on the gut immune system, Willliams et al. [35], reported its effects on the neonatal development of gut mucosal T cells and myeloid cells in the mouse. Oral administrations of bifidobacteria strains have shown immune-enhancing effects [36]. Recent studies indicate shifts in the composition of the intestinal microbiota (increased numbers of facultative anaerobes, in conjunction with a decrease in beneficial organisms such as the anaerobic lactobacilli and bifidobacteria) were related with changes of the host's immune system reactivity [37].

In our work, the increase of the bifidobacteria population observed after PFM ingestion could beneficially affect the intestinal ecosystem by the many properties attributed to these bacteria. The immune-modulation effects that have been observed for bifidobacteria include: increased mucosal IgA production [38]; stimulation of phagocytic activity of mononuclear cells [39]; stimulation of natural killer cells activity [40]; increased lymphocyte responsiveness to oral and systemic challenge antigen [41,42]. In this sense, considering that the consumption of the PFM increased bifidobacteria population, some immunological parameters were measured to analyze the influence of the development of these bacteria on the regulation of the immune system.

The IgA+ cells in the lamina propria of the small intestine were evaluated because Moreau and Gaboriau-Routhiau [43] reported the importance of the complete microbiota establishment on the increase of the number of IgA+ cells. The role of the IgA+ cells in the intestine is undeniable [44]. The increase in this population was also induced by oral administration of a suspension of a probiotic bacterium *L. casei *CRL 431 [45] and by fermented milks such as yoghurt [27] or the PFM used in the present work when administered to adult mice [13].

In this study, when the number of IgA+ cells was determined in the small intestines of newborn mice, it was observed that PFM administration to their mothers had no influence during the breast feeding period, compared to the untreated control (Fig. 3). After weaning (28 and 45 days), newborns from the control group (Aa) showed a progressive increase of IgA+ cells due to the maturation of their own adaptative immune system. In contrast, mice from group B showed a lower count of IgA+ cells at day 28. This last result could be related to the protective effect provided by the acquisition of the passive immunity through breast feeding, which was reinforced by the administration of PFM to their mothers. At the end of the experiment, when the mice reached maturity, the effect of the consumption of PFM by the mothers was not observed; all groups showed similar values for these cells, independent of the consumption of PFM by the offspring.

During the suckling phase of development, luminal intestinal secretory IgA is provided predominantly by the colostrum and breast milk, whereas in postweaned mice, secretory IgA (S-IgA) is synthesized by the weanlings own adaptive immune system [46,47]. Maternal IgG antibodies enter the fetal circulation through the placenta, whereas IgA antibodies in milk remain largely within the human infant's gut where they can influence the intestinal flora [17].

When the effect of the PFM on the S-IgA was analyzed it was observed that the animals whose mothers were given PFM had high levels of total S-IgA in the small intestinal fluid on day 12 (Fig. 4). This result could be related with an increase of IgA in the breast milk when the mother consumed PFM. It is believed that maternal antibodies may have a suppressive effect on the development of mucosal immune response in their offspring, leading to a partially developed immune system at weaning [48]. Studies in mice nursing for a prolonged time have shown a reduced quantity of IgA in intestinal washing at 5 weeks of age compared to naturally weaned litters, suggesting an active role for maternal antibodies in delaying natural IgA responses [49]. Previous reports showed that the oral administration of probiotic bacteria suspensions or fermented milk bacteria increased the IgA+ cells in mucosal tissue distant to the intestine, such as bronchus or mammary glands [50]. At this point of mice development, the enhancement of S-IgA in milk and consequently in the gut, could be one of the factors that influence the intestinal microbiota. It was demonstrated that an intact maternal immune system promotes the diversification of the commensal microbiota in nursing mice depending on the age and region of the intestine [51].

The lack of increases in the IgA+ cells number and S-IgA in the small intestine after weaning for mice that received PFM differ when compared to other results which showed increases for these parameters when the mice were administered with the PFM [13]. These results could be related with the immaturity of the immune system considering that the previous results were obtained when adult mice (older than 45 days of age) were used.

Macrophages are an important cell population for the innate immune response and might also be involved in the regulation of acquired immune responses as was reported in the response against mouse hepatitis virus strain A59 [52]. It was reported that probiotic bacteria can exert their beneficial properties on the host immune system by activating these cells [53]. The marker F4/80 is present on the surface of a family of cells member of the mononuclear phagocyte system of mice. The expression of this antigen can be considered a specialized adaptive state rather than a separate lineage, which is higher in mature macrophages and its expression is required for regulatory T cell development [54].

Dendritic cells are known to be essential immune cells in innate immunity and in the initiation of adaptive immunity. These cells capture and transfer information from the outside world to the cells of the adaptive immune system. They are not only critical for the induction of primary immune responses, but may also be important for the induction of immunological tolerance, as well as for the regulation of the type of T cell-mediated immune response [55]. It is known that the shaping of adaptive immunity by innate immunity is dependent on dendritic cells unique cellular functions and dendritic cell-derived effector molecules such as cytokines and chemokines [56]. Intestinal dendritic cells were studied in our model because they are likely to regulate immunity to gut microbiota. IL-10 production by dendritic cells was significantly increased following stimulation with *Bifidobacteria longum*, but not after exposure to lipopolysaccharide or *Streptococcus faecium*. Hart et al., [57] studied several probiotic bacteria and showed that they differ in their immunomodulatory activity and influence polarization of immune responses at the earliest stage of antigen presentation by dendritic cells, being the most marked anti-inflammatory effects produced by bifidobacteria strains which up-regulated IL-10 production by dendritic cells. In this work, the 33D1 antibody was used to study dendritic cells as is explained in the Results section. The antigen recognized by 33D1 is an inhibitory receptor and is present on a subpopulation of dendritic cells. The lack of this receptor might suggest a gain in function; however, dendritic cells recognized by 33D1 are more effective for antigen presentation on the class II major histocompatibility complex, than on dendritic cells without this receptor [58]; thus the antigen that binds 33D1 antibody on dendritic cells, may reflect their maturation state.

In our study it was determined for both macrophages and dendritic cells in the lamina propria of the small intestine that the administration of PFM to the mothers induced a marked down regulation in their offspring on day 12. These findings could mean that the offspring would be protected from the passive immunity provided by the mother or that the influence of the different microbiota population favours the down regulation of the immune cell markers assayed at day 12. This fact would allow a complete and equilibrated bacterial colonization of the intestine. An increased activity of the immune cells involved in phagocytic activity and antigen presentation would not be beneficial for the host at this time of the development. This immunoregulatory effect was not observed in newborn mice from the control group without PFM administration. At day 45, when the mice reached their maturity and when the microbiota establishment in the intestine was complete, the consumption of PFM by the mice increased the number of macrophages and dendritic cells. Results observed for these adult mice agree with previous studies where the administration of probiotic bacteria can modulate the immune system and enhancing receptors related with the maturation of the gut associated immune cells [53]. These finding could be also related with other previous reports where the consumption of the PFM by adult mice stimulated the mucosal immune system with production of cytokines by not only from T cells but also from macrophages or dendritic cells [13].

The gastrointestinal epithelium is covered by a protective mucus containing predominantly mucin glycoproteins that are synthesized and secreted by goblet cells. Intestinal microbes may directly affect goblet cell functions through the local release of bioactive factors. Alternatively, goblet cell functions may be altered in response to host-derived bioactive factors generated by activated epithelial or underlying lamina propria cells after their contact with intestinal bacteria [59]. The concept of the mucus layer functioning as a dynamic defensive barrier is suggested by studies showing altered mucus-related indexes in germ-free animals [60,61] and from consistent evidence of enhanced mucus secretion in response to intestinal microbes [62].

The number of goblet cells in the groups of mice whose mothers received PFM during nursing decreased only in the first sample (12 days, Fig. 5); after which this cell population reached values similar to the control and maintaining them during all the experiment (independent of the consumption of PFM after weaning). After weaning, the effect of the consumption of PFM by the offspring was observed only in the mice from mothers that never received PFM (Ab). The observation that mice from mothers that received PFM did not show increases in these cells could be related with the passive immunity provide by the maternal immune system reinforced for the consumption of the PFM by their mothers as was explained for IgA+ cells.

The present work showed a postnatal modulation of the intestinal microbiota of the offspring influenced by consumption of fermented milk containing *L. casei *DN-114001 by their mothers during nursing and by the offspring after weaning. The administration of this fermented milk to the mothers during nursing improved their own immune system (as was reported previously in adult mice feeding with this PFM, [13]) and this was reflected in their offspring. The consumption of the PFM by the mothers or their offspring favored the growth of bifidobacteria which are related with the improvement of the gut immune system of the offspring. The down regulation observed during the suckling period could be related with the improvement of the immunity of the mother fed with the PFM, which passively protect the babies in this important period of their life. At weaning and one week afterwards is a critical period that could be compared to the first years of human babies where the immune system is maturing and it is desirable that the administration of a probiotic microorganism does not alter early innate immune responses in this population at high risk of developing allergic diseases [63]. At day 45, the mice reach the maturity of their own immune system and the effects observed in the mice that received PFM agree with previous works where adult mice were used and the PFM stimulated their mucosal immunity. Increases for secretory IgA of the babies was another tool by which the beneficial effect of PFM administration to the mother during breast feeding period can be explained.

## Conclusion

The main contribution of this work is the demonstration that the administration of a specific probiotic fermented milk during nursing has beneficial impact on the microbiota development of the nursing offspring and this was related with a modulation of two important immune cell populations (macrophages and dendritic cells) that are involved in both innate and acquired immunity.

The present work shows the beneficial effect of the administration of a probiotic fermented milk, not only to the mothers during the suckling period, but also to the offspring near and after weaning and in the adulthood where the immune system is matured and there are many reports about the beneficial effects of the probiotics on the immunity of the host. This effect was due to the improved balances of the intestinal microbiota which are related with a modulation of the intestinal immune response, which was observed with the stimulation of the IgA + cells, macrophages and dendritic cells.

## Abbreviations

PFM: probiotic fermented milk; S-IgA: secretory Immunoglobuline A; GIT: gastrointestinal tract; CFU: colony-forming unit; RCA: Reinforced clostridia agar; MRS: Mann-Rogosa-Sharp; PBS: phosphate buffered saline.

## Authors' contributions

AdMdL, CAD and CMG carried out the microbiological work and the animal studies. GP and EC conceived of the study. AdMdL, CAD, CMG and GP designed the experiments. AdMdL, CAD performed the statistical analyses and prepared the figures. AdMdL, CAD and GP wrote the draft of the manuscript. EC, RW revised it for significant intellectual content. All authors read and approved the final version of the manuscript.
